# Evidence for postnatal neurogenesis in the human amygdala

**DOI:** 10.1038/s42003-022-03299-8

**Published:** 2022-04-19

**Authors:** Sebastian S. Roeder, Petra Burkardt, Fabian Rost, Julian Rode, Lutz Brusch, Roland Coras, Elisabet Englund, Karl Håkansson, Göran Possnert, Mehran Salehpour, Daniel Primetzhofer, László Csiba, Sarolta Molnár, Gábor Méhes, Anton B. Tonchev, Stefan Schwab, Olaf Bergmann, Hagen B. Huttner

**Affiliations:** 1 Department of Neurology, University of Erlangen-Nuremberg, Erlangen, Germany; 2 Center for Regenerative Therapies (CRTD), TU Dresden, Dresden, Germany; 3 Max Planck Institute for the Physics of Complex Systems, Dresden, Germany; 4 Center for Information Services and High Performance Computing (ZIH), TU Dresden, Dresden, Germany; 5 Center for Molecular and Cellular Bioengineering, DRESDEN-concept Genome Center, TU Dresden, Dresden, Germany; 6 Department of Neuropathology, University of Erlangen-Nuremberg, Erlangen, Germany; 7 Department of Neuropathology, University of Lund, Lund, Sweden; 8 Tandem Laboratory, Uppsala University, Uppsala, Sweden; 9 Department of Physics and Astronomy, Uppsala University, Uppsala, Sweden; 10 Department of Neurology, Faculty of Medicine, University of Debrecen, Debrecen, Hungary; 11 MTA-DE Cerebrovascular and Neurodegenerative Research Group, Debrecen, Hungary; 12 Department of Pathology, University of Debrecen, Debrecen, Hungary; 13 Departments of Anatomy, Cell Biology and Stem Cell Biology, Medical University Varna, Varna, Bulgaria; 14 Department of Cell and Molecular Biology, Karolinska Institute, Stockholm, Sweden; 15 Department of Neurology, Justus Liebig University Giessen, Giessen, Germany

**Keywords:** Adult neurogenesis, Neural ageing

## Abstract

The human amygdala is involved in processing of memory, decision-making, and emotional responses. Previous studies suggested that the amygdala may represent a neurogenic niche in mammals. By combining two distinct methodological approaches, lipofuscin quantification and ^14^C-based retrospective birth dating of neurons, along with mathematical modelling, we here explored whether postnatal neurogenesis exists in the human amygdala. We investigated post-mortem samples of twelve neurologically healthy subjects. The average rate of lipofuscin-negative neurons was 3.4%, representing a substantial proportion of cells substantially younger than the individual. Mass spectrometry analysis of genomic ^14^C-concentrations in amygdala neurons compared with atmospheric ^14^C-levels provided evidence for postnatal neuronal exchange. Mathematical modelling identified a best-fitting scenario comprising of a quiescent and a renewing neuronal population with an overall renewal rate of >2.7% per year. In conclusion, we provide evidence for postnatal neurogenesis in the human amygdala with cell turnover rates comparable to the hippocampus.

## Abstract (web summary)

Lipofuscin labeling and ^14^ C retrospective birth-dating of neurons, along with mathematical modelling, here suggest continued postnatal neurogenesis in the human amygdala, rather than protracted maturation of developmentally generated neurons.

## Introduction

The existence of postnatal neurogenesis in mammals has been debated for decades among neuroscientists^1^. Various studies provided evidence for adult neurogenesis in rodents as well as non-human primates, notably in two neurogenic niches, i.e. the subgranular zone of the dentate gyrus of the hippocampus and the subventricular zone of the lateral ventricles^2,3^. These findings in the CNS of animals are widely accepted as neuronal cell turnover was verified by independent methodological approaches. Immunohistochemistry in combination with genomic labeling using BrdU provided solid evidence for DNA-synthesis and long-term survival of new-born neurons in these neurogenic areas^1^.

In humans, analysis of neuronal turnover for decades had been restricted to immunohistochemical studies, while evidence from genomic labeling approaches was missing. Postnatal adult human hippocampal neurogenesis was first established in 1998 using genomic labeling with BrdU in five patients with squamous cell carcinoma who underwent BrdU infusion as part of their treatment^4^. In 2013, and 2014 respectively, the hypothesis of adult human neurogenesis was further supported based on analysis of genomic incorporation of ^14^C-radiocarbon, which was released upon the above-ground nuclear bomb tests within the Cold War. Landmark studies by Spalding et al. and Ernst et al. verified lifelong neurogenesis within both neurogenic niches, the human hippocampus and the striatum including the subventricular zone^5,6^.

However, there is mounting evidence that other regions of the human brain also harbor the potential for postnatal neurogenesis with the amygdaloid complex being one of the main areas of interest. Presence of immature neurons and neuronal cell proliferation within the adult amygdala have been reported in rodents^7–9^ and non-human primates using BrdU labeling^10–12^. Yet, in humans uncertainty remains regarding the limitation of markers for immature neurons^13^ and the lack of evidence from genomic labeling studies in man.

The present study aimed to explore the existence and dynamics of adult neurogenesis in the human amygdala using two methodologically distinct approaches: ^14^C-radiocarbon-based retrospective birth dating of cells and analysis of lipofuscin deposition. Absence of lipofuscin has only been described in neurons of individuals under the age of 5 years^14^, whereas the adult human cortex is lacking lipofuscin-negative neurons^15^. By combining ^14^C-radiocarbon analysis and lipofuscin-quantification, along with bio mathematical modelling, we here provide evidence of adult neurogenesis and propose a model for neuronal cell turnover dynamics within the adult human amygdala.

## Results

### Lipofuscin-negative neurons in the adult human amygdala indicate a neuronal cell age younger than the individual

**Fig. 1:**
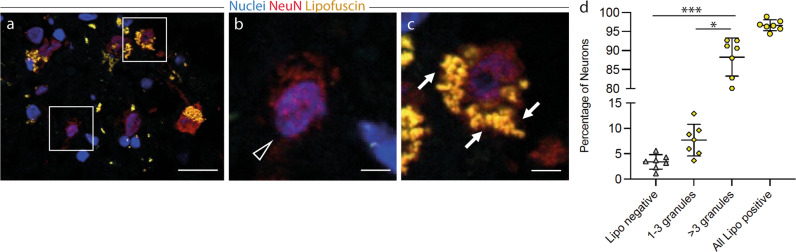
Lipofuscin-negative neurons in the adult human amygdala indicate a neuronal cell age younger than the individual. **a**–**d** Lipofuscin positive and negative neurons in the human amygdala. Immunofluorescence staining of tissue sections of the adult human amygdala. Neurons were identified by NeuN-staining (red) and nuclei were labeled with 4’,6-diamidino-2-phenylindole (DAPI, blue). Presence of lipofuscin granules (yellow) was assessed by autofluorescence signal after excitation at 488 nm. **a** Boxed areas from the respective overview image, **b** showing lipofuscin-negative neurons (arrow heads), **c** and lipofuscin-positive neurons (arrows). **d** Quantification of lipofuscin granules per neuron (*n* = 7 patients, quantification of an average of 234 neurons/patient). Proportions of trichotomized lipofuscin levels for subjects L1-L7 (Supplemental Table 1) are presented as mean ± SEM; Kruskal–Wallis One Way Analysis of Variance on Ranks with post-hoc Tukey: *p < 0.05; ***p < 0.001. **a** Scale bar = 10 µm, **b**, **c** scale bar = 2 µm.

Post-mortem amygdala tissue samples of seven neurologically healthy subjects aged between 50 and 86 years were analyzed (L1-L7, Supplementary Table 1). We quantified a mean of 234 neurons per subject using Z-stack image acquisition to examine the cell soma in its entirety (Supplementary Fig. 1). While 88.3% [84.6;92.0] of amygdaloid neurons showed abundant lipofuscin deposition (>3 granules) and 7.7% [5.4;10.0] had between one and three distinct lipofuscin granules, 3.4% [2.3;4.5] of neurons were devoid of any lipofuscin (Kruskal–Wallis One Way Analysis of Variance on Ranks, *H* = 15.95 with 2 degrees of freedom, *p* < 0.001) indicative of neurons with a cell age younger than the individual (Fig. 1 and Supplementary Figs. 1–3).

### Genomic ^14^C-levels of neuronal and non-neuronal cells in the adult human amygdala provide evidence for post-natal neurogenesis

**Fig. 2:**
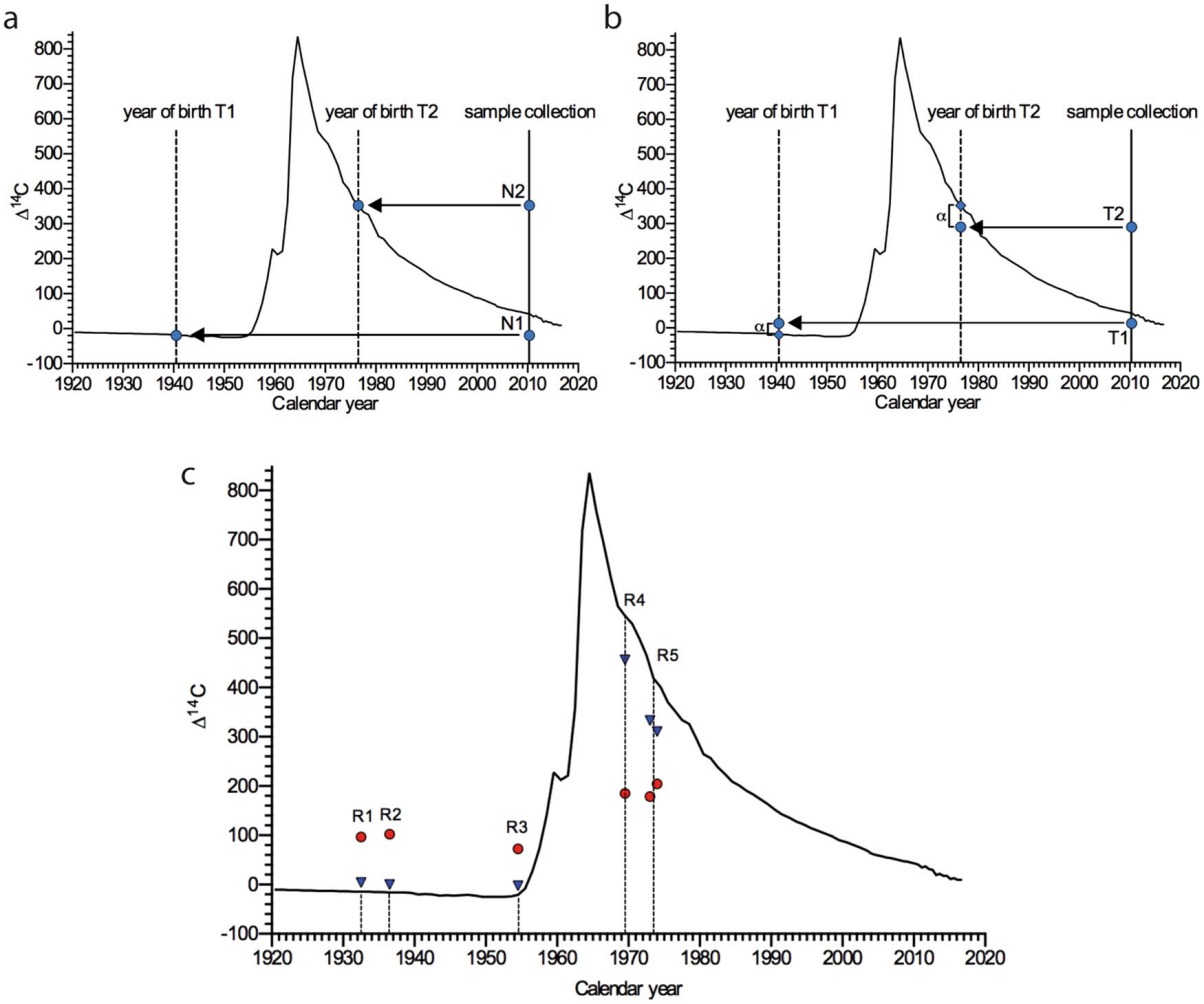
Genomic ^14^C-levels of neuronal and non-neuronal cells in the adult human amygdala provide evidence for post-natal neurogenesis. **a**, **b** Interpretation of two hypothetical scenarios of differential cell turnover utilizing radiocarbon-based retrospective birth dating of cells. Atmospheric ^14^C concentrations of the last century are depicted by the black line. The vertical solid line represents the year of hypothetical sample collection. **a** In a no-cell-turnover-scenario the concentrations of genomic ^14^C in the year of sample collection of two individuals (N1, born 1940 and N2, born 1976) are identical to the atmospheric ^14^C levels in the years of birth of the individuals. The vertical dashed line represents the year of birth of the individuals and in this scenario meets the atmospheric ^14^C curve at the exact ^14^C concentration found at sample collection. **b** In a cell-turnover scenario the concentrations of genomic ^14^C levels in the year of sample collection of two individuals (T1, born 1940 and T2, born 1976) differ (alpha) from the atmospheric ^14^C levels in the years of birth of the individuals (vertical dashed line). In this turnover scenario the ^14^C levels of individuals born before the ^14^C peak are higher than the atmospheric ^14^C curve in the year of birth, whereas the ^14^C levels of individuals born after the ^14^C peak are below the atmospheric ^14^C curve in the year of birth of individuals. **c** ^14^C concentrations determined by accelerator mass spectrometry of twelve DNA samples derived from the amygdalae of five different, neurologically healthy subjects (R1-R5; bilateral amygdala of patient R5). Individual values are plotted at the time of birth of the respective subject (vertical dashed line). ^14^C levels of non-neuronal cells (red) and neuronal cells (blue) differ from the atmospheric ^14^C levels at the time of birth of the individuals, indicating post-natal neurogenesis.

The radiocarbon-based retrospective birth dating approach and its interpretations are illustrated in Fig. 2a, b. To assess the age of human amygdaloid neurons we determined the ^14^C-concentration in the genome of five neurologically healthy subjects, aged between 44 and 86 years, by accelerator mass spectrometry in a post-mortem analysis (R1-R5, Supplementary Tables 1 and 2). Genomic ^14^C-concentrations of neuronal and non-neuronal cells of the amygdala are displayed in Fig. 2c. For subjects born before the atmospheric ^14^C-peak (R1-3) the genomic ^14^C-concentrations were above the atmospheric ^14^C-levels at the time of birth of the individual. In subjects born after the ^14^C-peak (R4-5) genomic ^14^C-concentrations were below atmospheric ^14^C-levels at the time of birth, indicating post-natal cell turnover.

### Dynamics of neuronal cell turnover in the adult human amygdala

**Fig. 3:**
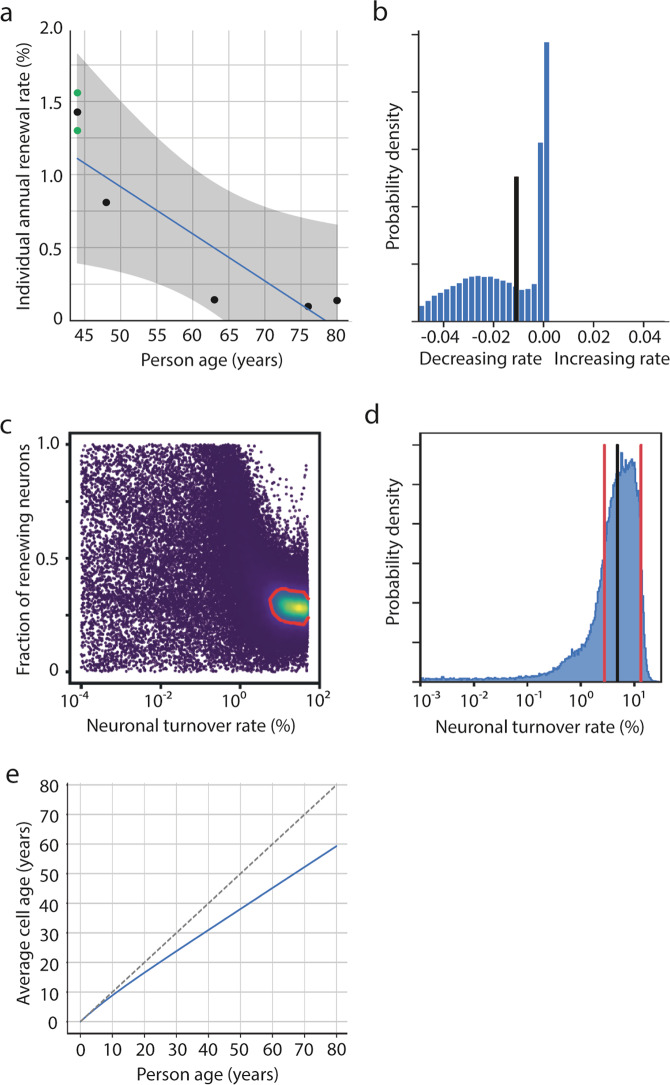
Dynamics of neuronal cell turnover in the human amygdala. Mathematical modelling of different neuronal cell turnover scenarios. **a** Scenario A assumes a constant turnover rate of neurons in the human amygdala over the lifetime of the individual. Fitting all samples to the model results in a median turnover rate of 0.2%/year [0.02;1.35]. However, separate fits for each sample revealed a negative correlation between age and individual turnover rate (*r* = −0.9, *p* = 0.083) with an annual decline in turnover of −0.03%/year. Green dots are amygdala measurements from both hemispheres from the same individual. Mean of these two dots are shown as black dot in between. **b** Scenario LIN, assuming a linear decline in cell turnover with age, revealed a median annual change in turnover rate (black vertical line) of −1 %/year [−4;0.08]. **c** Markov-Chain-Monte Carlo sampling for neuronal turnover for scenario 2POP, which assumes a quiescent and a renewing neuronal population, showing a two-dimensional marginal posterior distribution for turnover rate and fraction of renewing cells; blue: low probability, yellow: high probability, 1-sigma confidence region is framed by a red line. **d** Marginal posterior distribution for the estimated turnover rate calculated to the entire neuronal population, which is the product of the renewing rate of the renewing fraction, and the fraction of renewing cells, based on scenario POP2. Vertical red lines indicate lower and upper estimates (one sigma) of neuronal turnover. Black vertical line indicates median of turnover. **e** Age of amygdaloid neurons in relation to subject age based on the lower turnover limit of scenario 2POP. The bisecting (dashed) line represents a no-turnover-scenario in which every cell is as old as the individual, the blue line progressively deflects from the bisector with increasing subject age, reflecting lifelong post-natal neuronal cell turnover.

To investigate turnover dynamics of neurons in the human amygdala we employed bio mathematical modelling and fitted three different models to the dataset derived from ^14^C analysis. In Scenario A we assumed a constant cell turnover rate of the amygdaloid neurons over the lifetime of the individual. Bayesian parameter estimation using Markov-chain-Monte-Carlo (MCMC) sampling resulted in an estimation of an average turnover rate of 0.2%/year [0.02;1.35] (Supplementary Fig. 4). However, fitting individual turnover rates for each subject revealed a negative correlation between patient age and individual turnover estimates (*r* = −0.9, *p* = 0.083), suggesting a decline of neuronal cell renewal over time (−0.03%/year; Fig. 3a). Therefore, in a second step, scenario LIN was fitted to the data, modelling a linear change in turnover over time. This model estimated a median annual decline of 1%/year [−0.081;4] with a likelihood of 75% for a declining neuronal turnover rate (Fig. 3b) in the human amygdala over time (Supplementary Fig. 5).

Given the assumption that, similar to the human hippocampus, only a fraction of the neurons in the amygdala can be exchanged, a scenario 2POP was adapted that assumes a renewing and a resting neuron population in the human amygdala. Scenario 2POP was best suited to describe neuronal turnover dynamics in the adult human amygdala with a relative weight of 0.85 in a leave-one-out cross-validation (Supplementary Table 3). Parameter estimation by MCMC sampling resulted in a fraction of renewing cells of 0.29 [0.23;0.43] (Fig. 3c and Supplementary Fig. 6). Although we found high uncertainty in renewal parameter estimates in the POP2 scenario (Fig. 3c, Supplementary Table 3, and Supplementary Fig. 6), the lower limit of annual renewal calculated to the entire population was 2.7% per year (Fig. 3d), demonstrating robust adult neurogenesis in the human amygdala. The age-dependent estimates for average cell age of human amygdaloid neurons based on the lower limit of neurogenesis in this two-population-based scenario are displayed in Fig. 3e.

## Discussion

We here chose to utilize two methodologically distinct approaches to study cell turnover in the adult human amygdala. Both, analysis of neuronal lipofuscin deposition and ^14^C-radiocarbon-based determination of neuronal age, verified the presence of adult neurogenesis in the amygdaloid complex of the human brain. Bio mathematical modelling established presence of neurogenesis in the amygdala with a minimum turnover rate of 2.7% per year, based on the best-fitting 2POP scenario. Some aspects emerge from the data.

Regarding methodological approaches, existing evidence hinting towards existence of amygdaloid neurogenesis in rodents and non-human primates has been derived mainly from genomic BrdU-labeling experiments^10–12^. On the contrary, in humans, studies so far were restricted to immunohistochemical stainings for markers of immature neurons including doublecortin (DCX) and polysilated neural cell adhesion molecule (PSA-NCAM) leaving room for uncertainty^13,16^. Labeling of immature interneurons has led to conflicting results in the human hippocampus^17–20^. One explanation for this discrepancy is the variety of external factors such as post-mortem interval, tissue sample storage as well as duration and type of fixation that can compromise the validity of DCX/PSA-NCAM immunolabeling^18,21^. Moreover, expression of DCX and PSA-NCAM is not always restricted to immature neurons. DCX can also be found in postmitotic neurons in the piriformis cortex^22^, and DCX + progenitors can give rise to oligodendrocytes in pathological conditions^23^. Although PSA-NCAM is expressed together with DCX in immature cells in various species, it is also described in non-renewing interneurons and is therefore not only found in neurogenesis^24–26^. Thus, given the small sample size of our study, and the uncertainty of surrogate markers in the amygdala, we focused on two different approaches to establish the age of neurons in the human amygdala.^5,6,15^.

The accumulation of lipofuscin is not exclusively linked to aging, but can be also attributed to oxidative stress in pathological conditions^27^. However, the finding of a small fraction of neurons devoid of the age pigment lipofuscin in all investigated adult human amygdalae, in the absence of neurological or psychiatric disorders, hints towards presence of neurons with a cell age younger than that of the individual^14,15^. In addition, using restrospective ^14^C birth dating, a technique that generated unique insights into neuronal cell turnover dynamics in humans^5,6^, we demonstrated that amygdaloid neurons are younger than the individual, with a decreasing turnover rate over the lifetime of the individual. Our findings are in line with previously published reports describing a volumetric growth of the human amygdala during adolescence, paralleled by an increase in neuronal cell number^28,29^. Furthermore, a subset of immature DCX + /PSA-NCAM + neurons was recently characterized persisting into old age with a certain decline over time^30^. Taken together, the data provided here, strengthen the concept of neuronal turnover within the adult human amygdala under physiological conditions.

The amygdala - as part of the limbic system involved into processing of memory, decision-making and emotional responses - is closely interconnected with the hippocampus both by anatomical and functional projections^31^. The hippocampus formation is the *one* region in the human brain where existence of neurogenesis is least disputed^21^. Similar to the landmark study by Spalding et al. utilizing ^14^C radiocarbon analysis for the determination of hippocampal turnover dynamics^5^, we here demonstrate post-natal turnover of neurons in the human amygdala based on a quiescent and a cycling neuronal population with a comparable fraction of renewing cells (40% vs. 35%). Whether or not our data comprise clinical implications needs to be established in future studies. However, perturbations in the normal ontogenetic development of the human amygdala have been linked to neuropsychiatric and neurodevelopmental disorders^32^. Several studies described an impairment in human neurogenesis coinciding with pathologic conditions, e.g. Alzheimer’s and Huntington’s disease^6,18^. In light of the results published by Avino et al. demonstrating a lack of neuron number expansion during adolescence in patients with autism, one might speculate about a possible link to impaired neurogenesis in the human amygdala^29^.

Given the inherent limitations of immunohistochemical studies applying immature neuronal markers on one hand, and mounting evidence for the generation of postnatal neurons by retrospective ^14^C birth dating, it becomes evident that new single-cell-based techniques are needed to further advance the understanding of adult human neurogenesis. We believe that studies utilizing stable isotope-labeled nucleosides in humans, such as ^15^N-thymidine^33^, are likely to represent a valuable approach to prospectively study cell turnover at the single-cell level in man. Such a prospective in vivo labeling of novel DNA-synthesis, with subsequent post-biopsy mass spectrometry analysis, harbors the potential to unequivocally clarify the existence, or absence, of adult human neurogenesis both in physiological and pathological conditions.

We acknowledge several limitations of the study. A major limitation is the small number of subjects and the complexity of the carbon-dating approach. Establishing latter required substantial loss of amygdala cases, and carbon-dating itself prevented to save sufficient tissue for immunohistological analyses including studies of neuronal subtypes in various parts of the amygdala. Given the small sample size, the precision of the mathematical turnover scenarios is limited. Therefore, we report the minimum turnover rate based on the best-fitting scenario to provide a robust, conservative lower bound for neurogenesis in the amygdala. Due to the minimum number of cells required for reliable ^14^C measurement by accelerator mass spectrometry, a differential analysis of turnover in sub-regions of the amygdala, notably the paralaminar or basolateral nucleus^30^, was not feasible. Several studies hint towards a maximum in post-natal neurogenesis during childhood and adolescence^17,30^. While of particular interest, those post-mortem samples were not available for inclusion into our study. Moreover, analysis of lipofuscin may only serve as a surrogate given the influence of age and stimuli such as oxidative stress to its aggregation. In addition, this study did not address regional heterogeneity in lipofuscin distribution in different neuronal subtypes. Finally, and on a general note, human studies on postnatal neurogenesis should adhere to rigorous standards of patient selection (Boldrini et al.^19^) to reduce conflicting reports in the future.

In summary, we provide evidence for postnatal neurogenesis in the human amygdala in a similar magnitude as suggested to exist within the human hippocampus.

## Methods

### Patient selection and tissue collection

A total of 12 deceased patients (aged between 44 and 86 years) undergoing autopsy in the Department of (Neuro-)Pathology at the Universities of Lund, Sweden, Varna, Bulgaria, and Debrecen, Hungary were included in the study. Ethical approval was granted by all institutional and regional ethics committees based on the central ethic votes from Erlangen, Germany (104_13 B & 331_14 B). Patients were selected only if medical chart review verified absence of previous CNS disease, if death occurred unrelated to acute CNS injury, and consent was obtained from relatives in accordance with the Declaration of Helsinki. Clinical absence of primary CNS-disease involved any neurological or psychiatric disease as known or evident from medical charts of the subjects of this study, including medication for treatment of those diseases. Clinical management consisted of routine toxicological testing for CNS-active drugs, including alcohol, upon hospital admission as well as CNS-affecting system diseases such as chronic alcoholism, AIDS, history of resuscitation or mental retardation.

A coronal section through the Corpora mamillaria was undertaken to preserve orientation and to visualize the dentate gyrus and subventricular zone. The amygdala was then dissected avoiding any contamination of latter structures. The amygdaloid complex was identified and dissected by experienced neuropathologists. Tissue samples were then frozen and stored at −80 °C until further analysis. Details and characteristics of the patients are provided in Supplementary Table 1.

### Immunohistochemistry and lipofuscin quantification

For histological analysis the whole dissected and fresh frozen amygdaloid complex was cut in half in the coronal plane. Brain tissue was sectioned at 10 µm on a cryostat (Leica CM3050 S) and post-fixed with 4% PFA (wt/vol) buffered in PBS for 20 min. Sections were washed three times in TBS. For staining of mature neurons, sections were incubated overnight at room temperature with mouse monoclonal antibody to NeuN (1:500; clone A-60; Merck Millipore). After several washes donkey anti-mouse Alexa®568-conjugated secondary antibody (1:500; LifeTechnologies A10037) was applied and incubated for 1 h at room temperature. Both primary and secondary antibodies were diluted in blocking solution (3% donkey serum, 0,3% Triton-100 in TBS). To visualize cell nuclei, sections were stained with DAPI (1:10,000), and mounted after several TBS rinses using Prolong Gold® anti-fade reagent (Invitrogen). Histological samples were analyzed using a Zeiss Confocal Microscope LSM 780 and a Zeiss-Observer Z1 microscope equipped with an ApoTome (Zeiss) for Z-Stack acquisition. For Lipofuscin quantification a 1200 × 900 µm region of interest (Supplementary Fig. 3, red frame) in the central area of the coronal section of the amygdala was defined, representing the basolateral nuclei complex. Each neuron within the ROI underwent Z-Stack imaging as shown in Supplementary Fig. 1 to screen the whole cell for lipofuscin deposition. Lipofuscin granules were identified by autofluorescence after excitation at 488 nm. The burden of lipofuscin deposition was trichotomized into abundant deposition (>3 granules/neuron), low deposition (1–3 granules/neuron), and absence of lipofuscin (0 granules/neuron), as previously described^6^.

### Nuclei isolation and FACS

As described previously^15^, tissue samples were thawed and Dounce homogenized in 9.7 ml of lysis buffer (0.32 M sucrose, 5 mM CaCl_2_, 3 mM magnesium acetate, 0.1 mM Na_2_ EDTA, 10 mM Tris (pH 8.0), and 0.1% TritonX-100). The homogenized samples were suspended in 18.5 ml of sucrose solution (1.7 M sucrose, 10 mM Tris (pH 8.0), 3 mM sodium acetate), and layered onto a cushion of 9.3 ml sucrose solution. The samples were centrifuged at 26,000×*g* for 2.25 h at 4 °C. The pellet containing the isolated nuclei was resuspended in nuclei storage buffer (15% sucrose (wt/vol); 70 mM KCl, 2 mM MgCl_2_, 10 mM Tris pH 7.2). Isolated nuclei were stained with mouse monoclonal antibody to NeuN (1:1000; clone A60; Merck Millipore) overnight at 4 °C. The NeuN antibody was directly conjugated to Alexa647 (Alexa Fluor 647 antibody labeling kit, Invitrogene). Prior to FACS analysis nuclei were centrifuged at 500×*g* for 10 min at 4 °C, resuspended in NBS-buffer (1% Sucrose (wt/vol), 70 mM KCl, 2 mM MgCl_2_, 10 mM Tris pH 7.2) and filtered using a 30 µm mesh nylon filter. Flow cytometry analyzes and sorting were performed using a MoFlo XDP instrument (Beckmann Coulter) at the Nikolaus-Fiebiger-Center of Molecular Medicine Erlangen. FACS gating strategy for sorts is shown in Supplementary Fig. 2.

### DNA purification and accelerator mass spectrometry

As described previously^15,34^, to prevent carbon contamination of the samples, glassware was prebaked for 8 h at 300 °C in an oven (L 5/11, Nabertherm) and all experiments were performed under a laminar flow hood (Hera safe KS 18, Thermo Scientific). Nuclei were filled up to a final volume of 1 ml with 500–800 µl lysis buffer (200 mM NaCl, 1% SDS (wt/vol), 5 mM EDTA, 100 mM Tris pH 8.0), and incubated with 8 µl Proteinase K (20 mg/ml; Invitrogene) overnight at 64 °C. In all, 3 µl of RNase-Cocktail (Invitrogene) were added and incubated for 1 h at 64 °C. Half of the existing volume of 5 M NaCl was added, agitated for 15 s and centrifuged for 3 min at 13,000×*g*. The supernatant was transferred to a 10-ml glass vial and three times the volume of ethanol was added to precipitate the DNA. The glass tube was inverted several times until the DNA strain became visible. The DNA precipitate was transferred to a glass dish and washed three times in DNA washing solution (70% ethanol (vol/vol), 0,1 M NaCl). The DNA was transferred to a glass vial, containing 200 µl of DNAse/RNAse free water and carefully dried at 64 °C. Finally, the DNA was dissolved in 500 µl DNAse/RNAse-free water. DNA quantity and purity were determined by UV spectroscopy (NanoDrop).

Accelerator mass spectrometry measurements were performed as described previously^15,35^. In brief, purified DNA samples suspended in water were lyophilized to dryness. For conversion of DNA samples into graphite, excess CuO was added to each dry sample. Afterwards tubes were evacuated and sealed with a high-temperature torch. The tubes were then placed in a furnace set at 900 °C for 30 min to combust all carbon to CO2. The evolved CO2 gases were cryogenically purified, trapped, and reduced to graphite in the presence of iron powder catalyst in individual sub-ml reactors at 550 °C for 6 h. Each graphite powder was subsequently pressed into an aluminum target holder. The targets were then measured using a commercial 170 kV Green MICADAS (IonPlus AG, Zurich, Switzerland) accelerator mass spectrometer at the Tandem Laboratory at Uppsala University. Corrections for background contamination introduced during sample preparation were made as described previously^36^. The uncertainty of the measurement was determined for each sample and ranged between ±12 34‰ (2 SD) decay-corrected Δ^14^C for the large sample and small samples (10 μg C), respectively. All ^14^C data are reported as decay-corrected Δ^14^C and are provided in Supplementary Table 2 and Supplementary Data 2 including impurity-corrected data and correction strategy.

### Statistics and reproducibility

For lipofuscin analysis data is presented as mean ± SEM; The significance was tested using Kruskal–Wallis One Way Analysis of Variance on Ranks with post-hoc Tukey: *p < 0.05; ***p < 0.001. The total number of individuals (*n*) is indicated in the text. For each biological replicate, 126 fields of view (1200 × 900 µm) were analyzed.

For ^14^C analysis the total number of individuals (*n*) is indicated in the text. One ^14^C measurements was performed for individuals R1 to R4. Two measurements were performed on individual R5, where both amygdalae were available. Estimated turnover rates based on mathematical modeling (see Statistical analysis and bio mathematical modelling of ^14^C data) from the ^14^C analysis are stated as mean and a 68% confidence interval.

### Statistical analysis and bio mathematical modelling of ^14^C data

Cell turnover dynamics for genetically labeled cell populations can be modeled with age-, concentration- and division-structured models^37–40^. In this study, we developed a concentration-structured model predicting ^14^C concentration dynamics from cell turnover rates. We describe the state of the cell population for a subject *i* with a density in ^14^C concentration space, where *n* is the density of cells with ^14^C concentration *c* at time *t* after birth of the subject. In our model we assume that newly generated cells incorporate the current atmospheric ^14^C concentration^15,41^. We modeled the dynamics utilizing the following population balance equation:

1 $$\frac{\partial {n}_{i}\left(c,t\right)}{{dt}}=\beta (t){N}_{0}\delta \left(c-{c}_{a}\left(t+b_i\right)\right)-\gamma (t){n}_{i}\left(c,t\right),$$

where *β*(*t*) is the birth rate, rescaled with the initial cell number *N*_0_. *δ*(*x*) reflects the Dirac delta function, *c*_*a*_(*t* + *b*) the atmospheric ^14^C concentration at time *t* after time of birth (*b*_*i*_), shifted by 1 year to account for delays due to the natural food chain, and *γ*(*t*) representing the cell death rate. Modelling homeostatic conditions, i.e. the total cell number *N*_*i*_(*t*) = ${\int_0^\infty} {dc}\; {ni}(c,t)$ is constant, neuronal cell turnover dynamics were based on one turnover rate *β*(*t*) = *γ*(*t*), upon which the following ordinary differential equation for dynamics of the mean ^14^C concentrations (c̅) was derived:

2 $${\bar{c}}_{i}\left(t\right)=\frac{{\int }_{0}^{{{\infty }}}{dc}\,c\,{n}_{i}\left(c,t\right)}{{N}_{i}\left(t\right)}:\frac{d{\bar{c}}_{i}\left(t\right)}{{dt}}=\beta (t)\left({c}_{a}\left(t+{b}_{i}\right)-{\bar{c}}_{i}\left(t\right)\right).$$

Together with the assumption that all cells incorporate the current atmospheric concentration at the time of birth, i.e. ${\bar{c}}_{i}(t=0)={c}_{a}({b}_{i})$, this fully specifies the dynamics of ^14^C concentration. We implemented a numerical solution for ^14^C concentration dynamics in Python (https://github.com/rodjul42/pyC14). We studied different variants of the model which we term scenarios: In scenario A, the turnover rate $\beta \left(t\right)=\beta$ is constant. In scenario LIN, cell turnover changes linearly over time, $\beta \left(t\right)={\beta }_{0}+\frac{{\beta }_{10}-{\beta }_{0}}{10{{\mbox{years}}}}t$. Here, ${\beta }_{0}$ and ${\beta }_{10}$ are the turnover rates at the age of 0 years and 10 years, respectively. To exclude negative and extreme turnover rates, the turnover rate is set to 0 if ${\beta }_{0}+\frac{{\beta }_{10}-{\beta }_{0}}{10{{\mbox{years}}}}t \; < \; 0$ and it is set to 0.5 if ${\beta }_{0}+\frac{{\beta }_{10}-{\beta }_{0}}{10{{\mbox{years}}}}t$ > 0.5. Finally, in Scenario 2POP, a fraction of cells *f* is renewing with rate $\beta$, while the other fraction of cells (*1-f*) is quiescent.

For parameter estimation and selection of the best-fitting model we utilized an additive Gaussian noise model to determine the likelihood of measured ^14^C concentrations, ${c}_{i}$ given the model parameters p

3 $${{{{{\mathcal{L}}}}}}\left({c}_{i},|,p\right)=\mathop{\prod }\limits_{i=1}^{n}\frac{1}{\sigma \sqrt{2\pi }}{e}^{-\frac{1}{2}{\left(\frac{{c}_{i}-{\bar{c}}_{i}\left({d}_{i}-{b}_{i}\right)}{\sigma }\right)}^{2}}$$

where ${d}_{i}$ is the date of sample collection for a subject *i* and *n* is the number of subjects. We assume that this Gaussian noise contains contributions from actual variability from subject to subject as well as measurement errors, and that the amplitude of the noise, given by the variance ${\sigma }^{2}$, is constant for all samples. We estimated the parameters using Bayesian inference and used a uniform prior in log-space for the turnover rates:

4 $${{\log }}\;\beta ,{{\log }}\;{\beta }_{0},{{\log }}\;{\beta }_{10}\sim{{{{{\mathcal{U}}}}}}\left({{\log }}\left({10}^{-6}\,{{{\mbox{year}}}}^{-1}\right),\,{{\log }}\left(0.5\,{{{\mbox{year}}}}^{-1}\right)\right)$$

and uniform priors for *f* and *s*:

$$f\sim{{{{{\mathcal{U}}}}}}\left(0,1\right),$$

$$s \sim {{{{{\mathcal{U}}}}}}\left(0{{\mbox{years}}},100{{\mbox{years}}}\right).$$

To numerically solve the Bayes theorem, we made use of Markov-Chain-Monte-Carlo (MCMC) sampling as described previously by Foreman-Mackey et al.^42^. For each scenario, we sampled chains with 2000 steps after a 1000 step burn-in. The number of chains is 50 times the number of parameters. The initial values for the unknown parameters were drawn from the prior distribution. A point estimate for parameter values was obtained from the posterior distribution (Supplementary Figs. 3–5) by calculating the median of the marginal distribution for each parameter.

For model selection, i.e. to select the scenario with the highest predictive power, we employed the leave-one-out (LOO) cross-validation which we computed using Pareto-smoothed importance sampling as implemented in ArviZ^43–45^. This also provides weights for each scenario which can be interpreted as the probability for each scenario to be the best-fitting one (Supplementary Table 3).

### Reporting summary

Further information on research design is available in the Nature Research Reporting Summary linked to this article.

## Supplementary information

Supplementary Material

Supplementary Data 1

Supplementary Data 2

Reporting Summary

## Data Availability

The datasets analyzed during the current study are available as Supplementary Data 1, and ^14^C data as Supplementary Data 2.

## Code availability

The code for parameter estimation is available online (https://github.com/fbnrst/roeder_et_al_2022).

## References

[1] Oppenheim RW (2019). Adult hippocampal neurogenesis in mammals (and humans): the death of a central dogma in neuroscience and its replacement by a new dogma. Dev. Neurobiol..

[2] Altman J, Das GD (1965). Autoradiographic and histological evidence of postnatal hippocampal neurogenesis in rats. J. Comp. Neurol..

[3] Gould E, Reeves AJ, Graziano MS, Gross CG (1999). Neurogenesis in the neocortex of adult primates. Science.

[4] Eriksson PS (1998). Neurogenesis in the adult human hippocampus. Nat. Med..

[5] Spalding KL (2013). Dynamics of hippocampal neurogenesis in adult humans. Cell.

[6] Ernst A (2014). Neurogenesis in the striatum of the adult human brain. Cell.

[7] Shapiro LA, Ng K, Zhou QY, Ribak CE (2009). Subventricular zone-derived, newly generated neurons populate several olfactory and limbic forebrain regions. Epilepsy Behav..

[8] Nacher J, Lanuza E, McEwen BS (2002). Distribution of PSA-NCAM expression in the amygdala of the adult rat. Neuroscience.

[9] Fowler CD, Liu Y, Wang Z (2008). Estrogen and adult neurogenesis in the amygdala and hypothalamus. Brain Res. Rev..

[10] Bernier PJ, Bedard A, Vinet J, Levesque M, Parent A (2002). Newly generated neurons in the amygdala and adjoining cortex of adult primates. Proc. Natl Acad. Sci. USA.

[11] Zhang XM (2009). Doublecortin-expressing cells persist in the associative cerebral cortex and amygdala in aged nonhuman primates. Front. Neuroanat..

[12] Marlatt MW (2011). Distinct structural plasticity in the hippocampus and amygdala of the middle-aged common marmoset (Callithrix jacchus). Exp. Neurol..

[13] Marti-Mengual U, Varea E, Crespo C, Blasco-Ibanez JM, Nacher J (2013). Cells expressing markers of immature neurons in the amygdala of adult humans. Eur. J. Neurosci..

[14] Benavides SH, Monserrat AJ, Fariña S, Porta EA (2002). Sequential histochemical studies of neuronal lipofuscin in human cerebral cortex from the first to the ninth decade of life. Arch. Gerontol. Geriatr..

[15] Huttner HB (2014). The age and genomic integrity of neurons after cortical stroke in humans. Nat. Neurosci..

[16] Ulfig N, Chan WY (2004). Expression patterns of PSA-NCAM in the human ganglionic eminence and its vicinity: role of PSA-NCAM in neuronal migration and axonal growth?. Cells Tissues Organs.

[17] Sorrells SF (2018). Human hippocampal neurogenesis drops sharply in children to undetectable levels in adults. Nature.

[18] Moreno-Jimenez EP (2019). Adult hippocampal neurogenesis is abundant in neurologically healthy subjects and drops sharply in patients with Alzheimer’s disease. Nat. Med..

[19] Boldrini M (2018). Human hippocampal neurogenesis persists throughout aging. Cell Stem Cell.

[20] Knoth R (2010). Murine features of neurogenesis in the human hippocampus across the lifespan from 0 to 100 years. PLoS ONE.

[21] Kempermann G (2018). Human adult neurogenesis: evidence and remaining questions. Cell Stem Cell.

[22] Klempin F, Kronenberg G, Cheung G, Kettenmann H, Kempermann G (2011). Properties of doublecortin-(DCX)-expressing cells in the piriform cortex compared to the neurogenic dentate gyrus of adult mice. PLoS ONE.

[23] Klein B (2020). DCX(+) neuronal progenitors contribute to new oligodendrocytes during remyelination in the hippocampus. Sci. Rep..

[24] La Rosa C, Parolisi R, Bonfanti L (2020). Brain structural plasticity: from adult neurogenesis to immature neurons. Front. Neurosci..

[25] Gilabert-Juan J, Castillo-Gomez E, Perez-Rando M, Molto MD, Nacher J (2011). Chronic stress induces changes in the structure of interneurons and in the expression of molecules related to neuronal structural plasticity and inhibitory neurotransmission in the amygdala of adult mice. Exp. Neurol..

[26] Gomez-Climent MA (2008). A population of prenatally generated cells in the rat paleocortex maintains an immature neuronal phenotype into adulthood. Cereb. Cortex.

[27] Brunk UT, Terman A (2002). Lipofuscin: mechanisms of age-related accumulation and influence on cell function. Free Radic. Biol. Med..

[28] Schumann CM (2004). The amygdala is enlarged in children but not adolescents with autism; the hippocampus is enlarged at all ages. J. Neurosci..

[29] Avino TA (2018). Neuron numbers increase in the human amygdala from birth to adulthood, but not in autism. Proc. Natl Acad. Sci. USA.

[30] Sorrells SF (2019). Immature excitatory neurons develop during adolescence in the human amygdala. Nat. Commun..

[31] Pare D, Dong J, Gaudreau H (1995). Amygdalo-entorhinal relations and their reflection in the hippocampal formation: generation of sharp sleep potentials. J. Neurosci..

[32] Schumann CM, Bauman MD, Amaral DG (2011). Abnormal structure or function of the amygdala is a common component of neurodevelopmental disorders. Neuropsychologia.

[33] Steinhauser ML (2012). Multi-isotope imaging mass spectrometry quantifies stem cell division and metabolism. Nature.

[34] Huttner HB (2018). Meningioma growth dynamics assessed by radiocarbon retrospective birth dating. EBioMedicine.

[35] Salehpour M, Håkansson K, Possnert G (2013). Accelerator mass spectrometry of ultra-small samples with applications in the biosciences. Nucl. Instrum. Methods Phys. Res. Sect. B Beam Interact. Mater. At..

[36] Salehpour M, Hakansson K, Possnert G, Wacker L, Synal HA (2016). Performance report for the low energy compact radiocarbon accelerator mass spectrometer at Uppsala University. Nucl. Instrum. Methods Phys. Res. Sect. B Beam Interact. Mater. At..

[37] Bernard S, Frisén J, Spalding KL (2010). A mathematical model for the interpretation of nuclear bomb test derived 14 C incorporation in biological systems. Nucl. Instrum. Methods Phys. Res. Sect. B Beam Interact. Mater. At..

[38] Hasenauer, J., Schittler, D. & F., Allgöwer F. Analysis and simulation of division- and label-structured population models: a new tool to analyze proliferation assays. *Bull. Math. Biol.* **74**, 2692–732 (2012). 10.1007/s11538-012-9774-5 23086287

[39] Hross, S. & Hasenauer, J. Analysis of CFSE time-series data using division-, age- and label-structured population models. *Bioinformatics* **32**, 2321–2329 (2016). 10.1093/bioinformatics/btw131 27153577

[40] Schittler D, Allgower F, De Boer RJ. A new model to simulate and analyze proliferating cell populations in BrdU labeling experiments. *BMC Syst Biol*. **7** Suppl 1, S4 (2013). 10.1186/1752-0509-7-S1-S4 PMC3750481 24268033

[41] Bergmann O (2012). The age of olfactory bulb neurons in humans. Neuron.

[42] Foreman-Mackey D, Hogg DW, Lang D, Goodman J (2013). emcee: the MCMC hammer. Publ. Astron. Soc. Pac..

[43] Vehtari A, Gelman A, Gabry J (2017). Practical Bayesian model evaluation using leave-one-out cross-validation and WAIC. Stat. Comput..

[44] Kumar R, Carroll C, Hartikainen A, Martin O (2019). ArviZ a unified library for exploratory analysis of Bayesian models in Python. J. Open Source Softw..

[45] Kumar A, Pareek V, Faiq MA, Ghosh SK, Kumari C (2019). Adult neurogenesis in humans: a review of basic concepts, history, current research, and clinical implications. Innov. Clin. Neurosci..

