# Supplementary material for: Evidence for postnatal neurogenesis in the human amygdala
Source: Commun Biol. 2022 Apr 19;5:366. doi: 10.1038/s42003-022-03299-8 (PMC9018740; doi:10.1038/s42003-022-03299-8)
Supplement: Supplementary file 1 — Supplementary Material [file 42003_2022_3299_MOESM1_ESM.pdf]

Supplementary Material

Evidence for Postnatal Neurogenesis in the Human Amygdala

Sebastian S. Roeder, Petra Burkardt, Fabian Rost, Julian Rode, Lutz Brusch,
Roland Coras, Elisabet Englund, Karl Håkansson, Göran Possnert, Mehran Salehpour, Daniel Primetzhofer, László Csiba, Sarolta Molnár, Gábor Méhes,
Anton B. Tonchev, Stefan Schwab, Olaf Bergmann*, Hagen B. Huttner*

Supplementary Table 1. Patient characteristics

Supplementary Table 2. $\Delta^{14}C$ values and related data of the included patients

Supplementary Table 3. Selection of best fitting model by leave-one-out cross validation

Supplementary Figure 1. Z-Stack image acquisition for lipofuscin quantification

Supplementary Figure 2. Representative images of Lipofuscin positive and negative neurons in the human Amygdala.

Supplementary Figure 3. Representative mosaic image of the human amygdala

Supplementary Figure 4. FACS-based isolation of neural nuclei from the human amygdala

Supplementary Figure 5. Bayesian parameter inference for scenario A

Supplementary Figure 6. Bayesian parameter inference for scenario LIN

Supplementary Figure 7. Bayesian parameter inference for scenario 2POP

Supplementary References.

* These authors contributed equally

Supplementary Table 1. Patient characteristics.

Pat-ID	*Year of birth*	*Sex*	*Age*	*Post mortem time (h)*	*Cause of death*	*Sample obtained*
R1	1932	f	80	30	Cardiorespiratory failure	Left Amygdala, Cortex
R2	1935	m	77	24	Pneumonia	Right Amygdala, Cortex
R3	1954	m	63	9	Trauma	Right Amygdala
R4	1969	m	48	4	Pulmonary Tuberculosis	Right Amygdala
R5	1973	m	44	12	Sepsis	Bilateral Amygdala
L1	1960	m	50	24	Cardiorespiratory failure	Amygdala
L2	1931	m	80	16	Cardiac Arrest	Amygdala
L3	1925	f	86	36	Pneumonia	Amygdala
L4	1954	f	57	22	Cardiac Arrest	Amygdala
L5	1960	m	51	18	Pneumonia	Amygdala
L6	1944	m	66	26	Pneumonia	Amygdala
L7	1953	m	58	32	Sepsis	Amygdala

The table provides details of the 12 patients included in the study. Samples of subjects R1-R5 were analysed by ^{14}C radiocarbon dating (bilateral amygdala samples of subject R5). Samples of subjects L1-L7 were analysed by immunofluorescence staining.

Supplementary Table 2. $\Delta^{14}C$ values and related data of patients R1-R5.

Sample-ID	Tissue	Postmortem time (h)	Sort	Δ14C (‰)	Δ14C (‰, 2 SD)	Carbon mass (µg DNA measured)	FACS purity (%)	Purity corrected Δ14C (‰)	Carbon mass (µg in reactor)
R1a	Amygdala, left	30	NeuN-	96,1	16	11,7	98,7	97,3	11,3
R1b	Amygdala, left	30	NeuN+	5,6	18	7,5	97,3	3,1	7,3
R2a	Amygdala, right	24	NeuN-	101,5	14	29,4	98,9	102,7	28,1
R2b	Amygdala, right	24	NeuN+	-0,8	22	6,9	98,0	-2,9	5,9
R3a	Amygdala, right	9	NeuN-	72,6	21	10,7	99,7	72,8	5,4
R3b	Amygdala, right	9	NeuN+	-1,9	13	23,7	98,2	-3,3	14,4
R4a	Amygdala, right	4	NeuN-	191,8	34	15,3	97,7	185,5	6,2
R4b	Amygdala, right	4	NeuN+	448,6	22	26,6	97,2	456,3	10
R5a	Amygdala, right	12	NeuN-	179,4	20	47,82	99,4	178,4	12,5
R5b	Amygdala, right	12	NeuN+	324,3	15	76,48	94,2	333,2	22,1
R5c	Amygdala, left	12	NeuN-	204,7	16	48,83	99,9	204,6	18,3
R5d	Amygdala, left	12	NeuN+	304,3	16	69,73	93,8	310,9	21,3

The table provides detailed results for $\Delta^{14}C$ radiocarbon dating and related data of patients R1-R5 (NeuN+=neuronal fraction, NeuN-=non-neuronal fraction, SD=standard deviation, FACS=Fluorescence Activated Cell Sorting).

Correction for impurities in FACS sorting was achieved by solving the following equation system for $\Delta^{14}C_{NeuN+}$ and $\Delta^{14}C_{NeuN-}$ with $x_{impurity\_NeuN+}$ and $y_{impurityNeuN-}$ displayed as percentage.

$$(1)\ \Delta 14C_{Nonneurons_{measured}} * 100 = \left(100 - y_{impurity_{Nonneurons}}\right) * \Delta 14C_{Nonneurons_{corrected}} + y_{impurity_{Nonneurons}} * \Delta 14C_{Neurons_{corrected}}$$

$$(2)\ \Delta 14C_{Neurons_{measured}} * 100 = \left(100 - x_{impurity_{Neurons}}\right) * \Delta 14C_{Neurons_{corrected}} + x_{impurity_{Neurons}} * \Delta 14C_{Noneurons_{corrected}}$$

Supplementary Table 3. Selection of best fitting model by leave-one-out cross validation.

rank	Scenario	loo	Weight	Parameter	Value	Confidence Interval
**0**	**x2POP**	**7.5**	**84%**	beta	17.00%/year	[10.748%/year - 49.997%/year]
				f	0.2927	[0.2335 - 0.3432]
				sigma	0.0316	[0.0132 - 0.0446]
**1**	**LinB**	**4.97**	**10%**	beta0	11.3%/year	[0.28%/year - 50.00%/year]
				beta10	0.0373%/year	[0.0013%/year - 0.8587%/year]
				sigma	0.0766	[0.0397 - 0.1010]
**2**	**A**	**4.62**	**6%**	beta	0.234%/year	[0.0245%/year - 1.3528%/year]
				sigma	0.0904	[0.0488 - 0.1159]

The table shows results of LOO cross validation and parameter estimations for scenarios A, LIN and 2POP (LOO=leave-one-out score, weight=relative predictive weight, β=turnover rate in %/year, f=fraction of renewing cells, σ=standard deviation of Gaussian noise model in %/year).

Supplementary Figure 1. Z-Stack image acquisition for lipofuscin quantification.

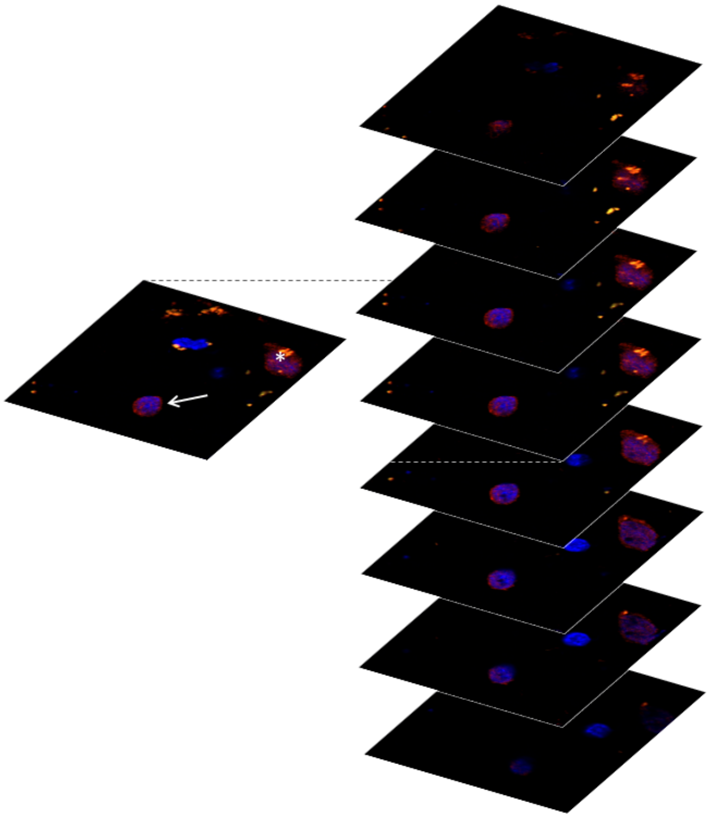

For quantification of lipofuscin granules, a Z-stack of immunofluorescence images was acquired for each cell, or cluster of cells, respectively. Presence or absence of lipofuscin was scored on each consecutive Z-stack image and the total number of lipofuscin granules (green) calculated for the whole cell. The asterisk marks a neuron with several lipofuscin granules, the arrow a neuron devoid of lipofuscin (see also Figure 1). Red color indicates NeuN labeling, Blue color indicates DAPI. Green lipofuscin granules appear yellow in the red NeuN overlays.

Supplementary Figure 2. Representative images of Lipofuscin positive and negative neurons in the human Amygdala.

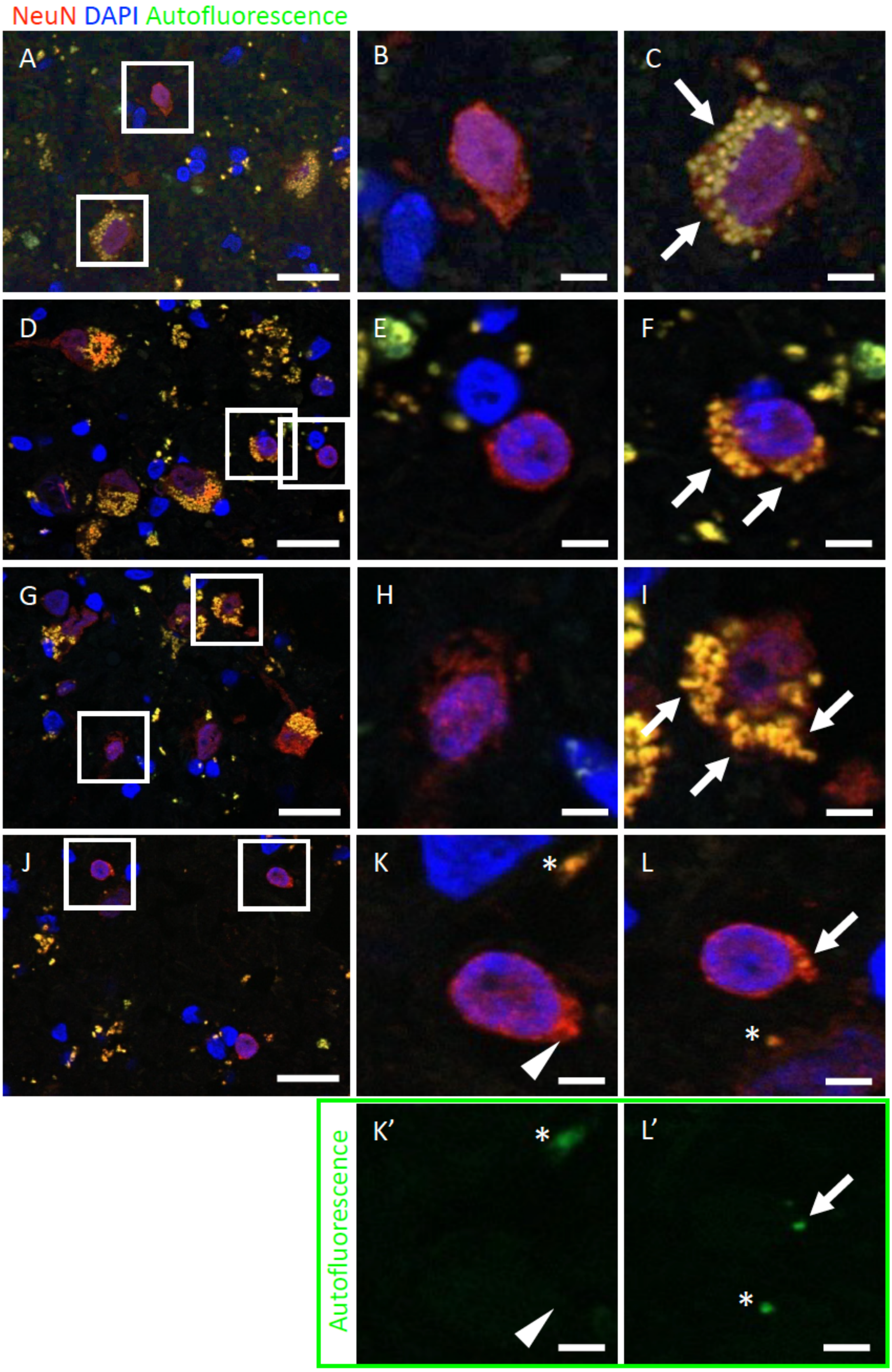

NeuN (red) / DAPI (blue) staining of human Amygdala sections used for Lipofuscin quantification. Lipofuscin granules were identified by autofluorescence signal at 488nm (green) and intracellular localization. The left row (A,D,G,J) shows overview images of the central amygdaloid complex. The middle (B,E,H,K) and right row (C,F,I,L) show the boxed areas from the respective overview image on the left at higher magnification. (B,E,H,K) Images in the middle row show Lipofuscin negative neurons. (C,F,I,L) Images in the right row show Lipofuscin positive neurons. The arrows depict various Lipofuscin granules. (K) Lipofuscin negative neuron. The asterisk shows an extracellular autofluorescence signal. The arrowhead points at the intracellular plasma of the neuron. (K') Same area as in K in the autofluorescence channel revealing the extracellular autofluorescence signal (*) but no intracellular signal (arrowhead). (L) Lipofuscin positive neuron. The asterisk shows an extracellular autofluorescence signal. The arrow pointing at an intracellular Lipofuscin granule. (L') Same area as in L in the autofluorescence channel revealing the extracellular autofluorescence signal (*) and the intracellular signal (arrow) representing a Lipofuscin granule. Scale bars: left row 10µm, middle and right row 2µm.

Supplementary Figure 3. Representative mosaic image of human amygdala.

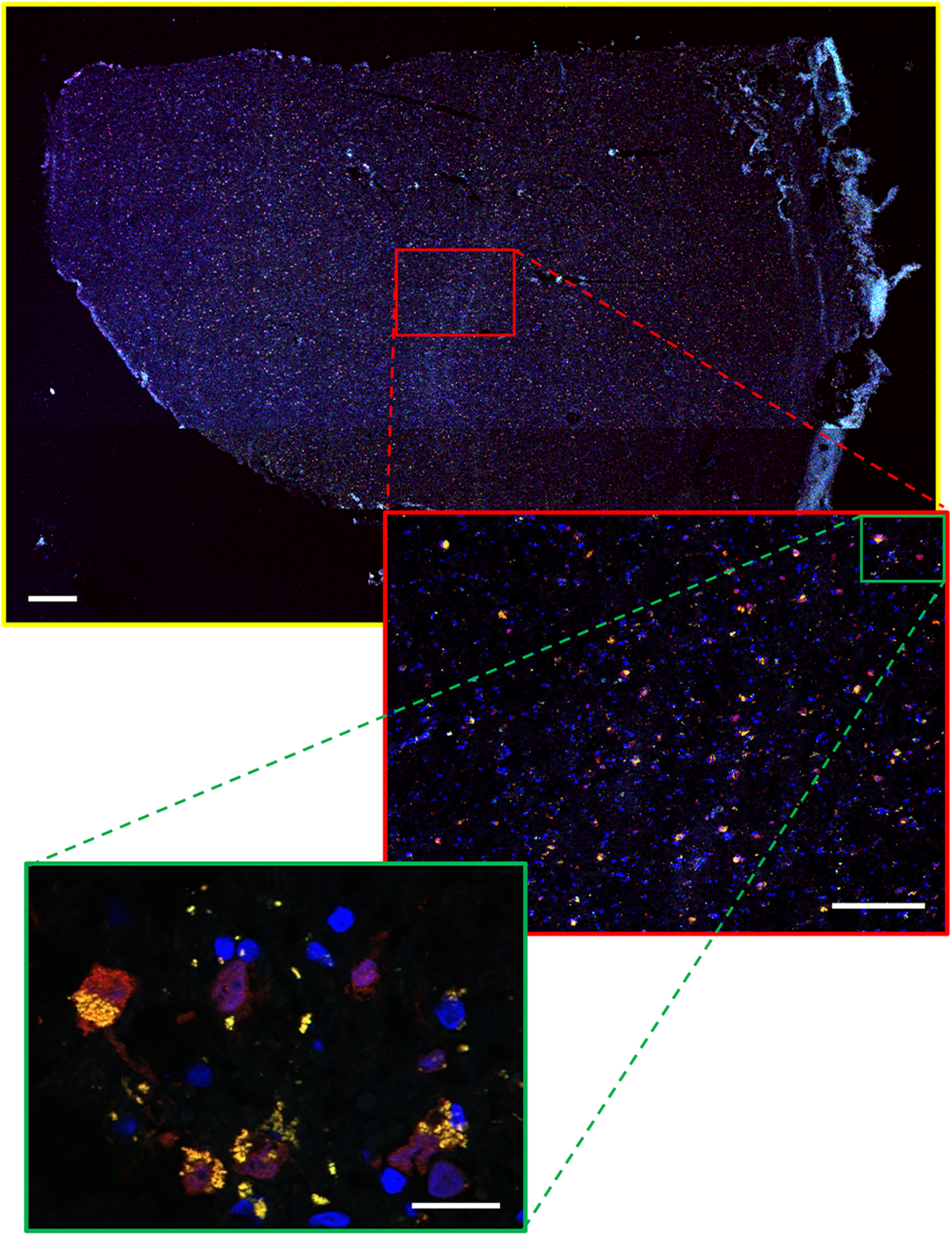

Mosaic image (yellow frame) of a representative amygdala sample used for lipofuscin quantification. The image consists of 126 (14x9) individual tiles acquired with a ZEISS confocal microscope. Immunofluorescence staining was performed for NeuN (red) / DAPI (blue). Lipofuscin granules (yellow) can be identified by autofluorescence signal at 488nm as described in Suppl. Fig. 2. For Lipofuscin quantification a 1200x900µm region of interest (red frame) in the central area of the dissected amygdala was defined, representing the basolateral

nuclei complex. Each neuron within the ROI underwent Z-Stack imaging as described in Suppl. Fig. 1 to screen the whole cell for lipofuscin deposition. The burden of lipofuscin deposition was trichotomized into abundant deposition (>3 granules/neuron), low deposition (1-3 granules/neuron) and absence of lipofuscin (0 granules/neuron). Scale bars: yellow frame 500µm, red frame 200µm, green frame 10µm.

Supplementary Figure 4. FACS-based isolation of neural nuclei from the human amygdala.

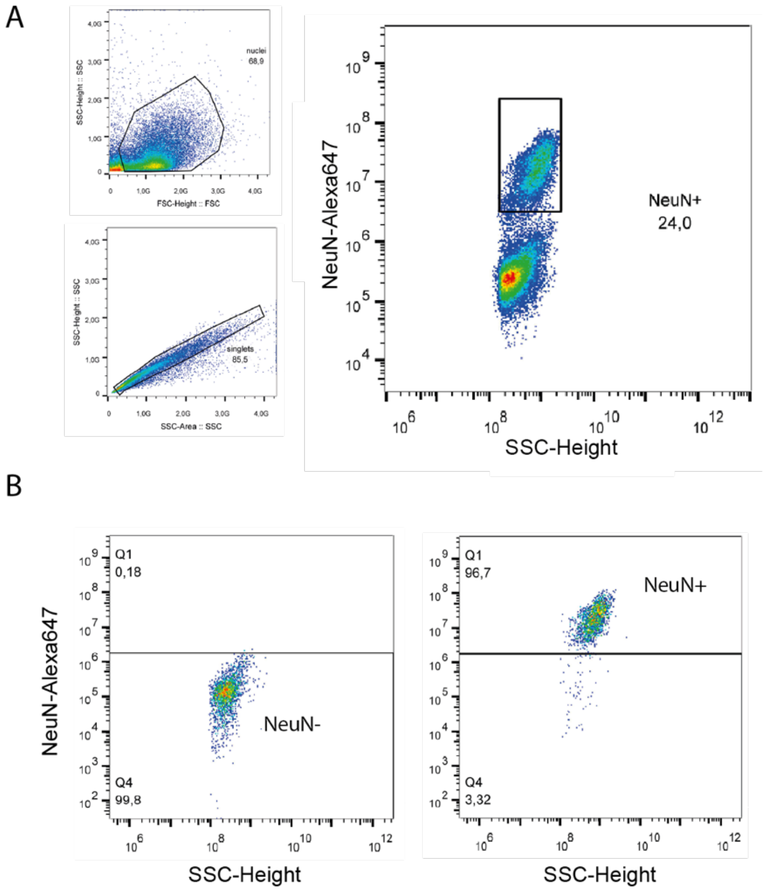

Representative FACS density plots of the isolation process. (A) Gating strategy of neuronal nuclei from human amygdala samples. Cell nuclei isolated from human amygdala samples were stained with anti-NeuN antibody and neuronal (black square) and non-neuronal cells sorted by flow cytometry. (B) Re-Analysis of sorted nonneuronal (NeuN-, left panel) and neuronal population (NeuN+, right panel) was performed for subsequent purity correction of the $\Delta^{14}C$ concentration (see Material and Methods section in main manuscript).

Supplementary Figure 5. Bayesian parameter inference for scenario A.

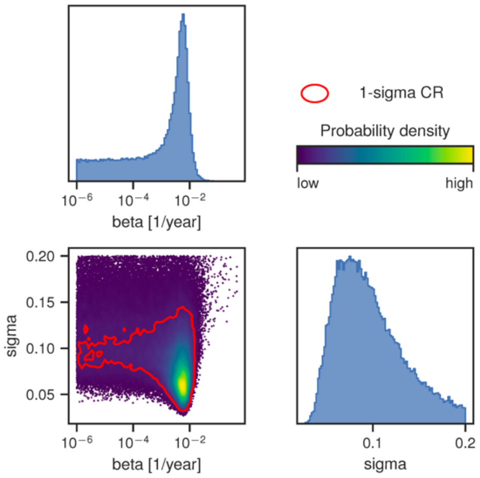

Results of parameter estimation by Markov-Chain-Monte-Carlo sampling for neuronal turnover in scenario A. The panels on the diagonal from upper left to lower right show marginal posterior distributions. The off-diagonal panel shows a two-dimensional marginal posterior distribution for a combination of two parameters on each panel (beta=turnover rate, sigma=standard deviation, blue=low probability, yellow=high probability, 1-σ confidence region is framed by a red line).

Supplementary Figure 6. Bayesian parameter inference for scenario LIN.

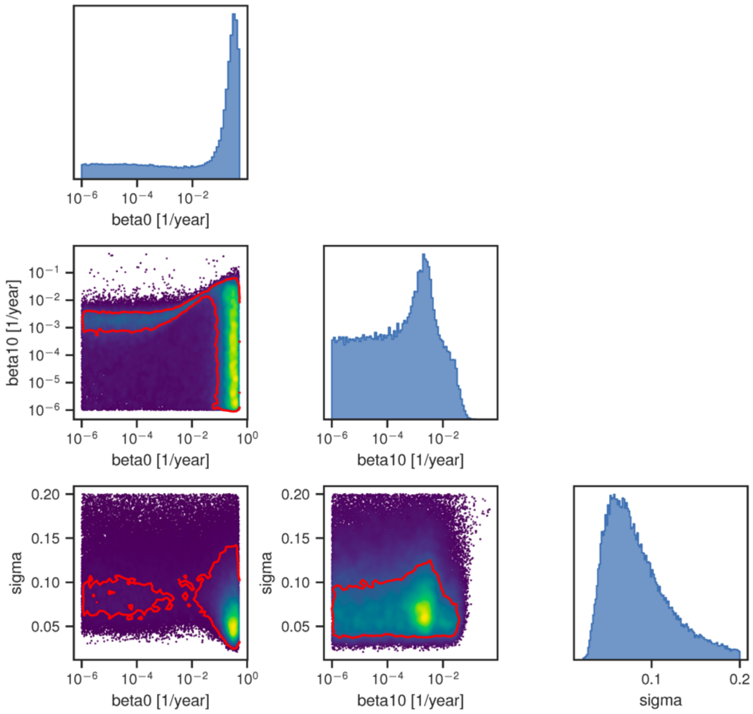

Results of parameter estimation by Markov-Chain-Monte-Carlo sampling for neuronal turnover in scenario LIN. The panels on the diagonal from upper left to lower right show marginal posterior distributions. The off-diagonal panels show a two-dimensional marginal posterior distribution for a combination of two parameters on each panel (beta=turnover rate, sigma=standard deviation, blue=low probability, yellow=high probability, 1-σ confidence region is framed by a red line).

Supplementary Figure 7. Bayesian parameter inference for scenario 2POP.

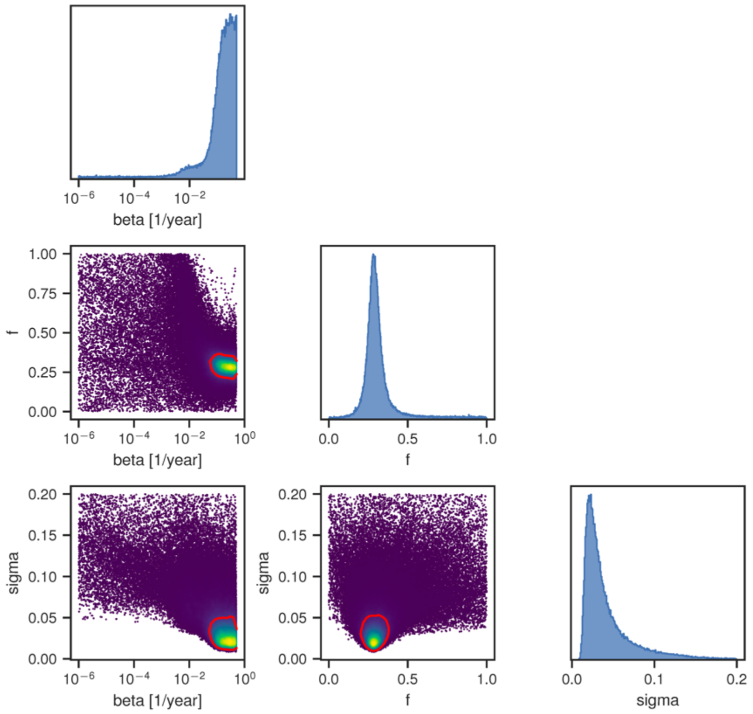

Results of parameter estimation by Markov-Chain-Monte-Carlo sampling for neuronal turnover in scenario 2POP. The panels on the diagonal from upper left to lower right show marginal posterior distributions. The off-diagonal panels show a two-dimensional marginal posterior distribution for a combination of two parameters on each panel (beta=turnover rate, sigma=standard deviation, f=fraction of renewing cells, blue=low probability, yellow=high probability, 1-σ confidence region is framed by a red line).
